# Effect of Surface Plasma Treatments on the Adhesion of Mars JSC 1 Simulant Dust to RTV 655, RTV 615, and Sylgard 184

**DOI:** 10.1371/journal.pone.0045719

**Published:** 2012-10-15

**Authors:** Firouzeh Sabri, Jeffrey G. Marchetta, M. Sinden-Redding, James J. Habenicht, Thien Phung Chung, Charles N. Melton, Chris J. Hatch, Robert L. Lirette

**Affiliations:** 1 Department of Physics, University of Memphis, Memphis, Tennessee, United States of America; 2 Department of Mechanical Engineering, University of Memphis, Memphis, Tennessee, United States of America; Harbin Institute of Technology, China

## Abstract

**Background:**

Dust accumulation on surfaces of critical instruments has been a major concern during lunar and Mars missions. Operation of instruments such as solar panels, chromatic calibration targets, as well as Extra Vehicular Activity (EVA) suits has been severely compromised in the past as a result of dust accumulation and adhesion. Wind storms with wind speeds of up to 70 mph have not been effective in removing significant amounts of the deposited dust. This is indeed an indication of the strength of the adhesion force(s) involved between the dust particles and the surface(s) that they have adhered to. Complications associated with dust accumulation are more severe for non-conducting surfaces and have been the focus of this work.

**Methodology:**

Argon plasma treatment was investigated as a mechanism for lowering dust accumulation on non-conducting polymeric surfaces. Polymers chosen for this study include a popular variety of silicones routinely used for space and terrestrial applications namely RTV 655, RTV 615, and Sylgard 184. Surface properties including wettability, surface potential, and surface charge density were compared before and after plasma treatment and under different storage conditions. Effect of ultraviolet radiation on RTV 655 was also investigated and compared with the effect of Ar plasma treatment.

**Conclusion/Significance:**

Gravimetric measurements proved Ar plasma treatment to be an effective method for eliminating dust adhesion to all three polymers after short periods of exposure. No physical damage was detected on any of the polymer surfaces after Ar plasma treatment. The surface potential of all three polymers remained zero up to three months post plasma exposure. Ultraviolet radiation however was not effective in reducing surface and caused damage and significant discoloration to RTV 655. Therefore, Ar plasma treatment can be an effective and non-destructive method for treating insulating polymeric surfaces in order to eliminate dust adhesion and accumulation.

## Introduction

Dust devils on planet surfaces such as Mars have caused irreversible damage and numerous complications for systems on board missions [1], [2], [3], [4], [5], [6]. Dust related hazards and concerns for equipment and crew is not limited to Mars and has been reported during lunar missions also [7], [8]. Addressing dust-related complications remains a challenge due to the different charging mechanisms [9], [10], [11], [12] each giving rise to a range of charge densities and polarities. The amount of dust settlement on exposed surfaces is primarily dominated by the materials' surface properties. Dust accumulation is of particular concern on non-conducting surfaces such as silicone-based components routinely used for encapsulation, vibration isolation, and electrical insulation purposes [13], [14].

Keeping critical surfaces clean is a crucial step towards proper operation of almost all systems and any surface contamination can lead to shortened lifetime of sensitive instruments. Since natural dust removal methods such as wind have not proven to be an effective cleaning method [2], other techniques must be investigated. On the other hand cleaning mechanisms that have moving parts are unattractive options due to the cost of maintenance, operation, and most importantly power limitations onboard space exploratory vehicles.

Currently, dust relief studies fall into three main categories: 1) Understanding dust grain charging mechanisms [8], 2) development of methods to remove settled dust [15], and 3) understanding surface-dust interactions. Development of methods that focus on dust removal after deposition is important but a more fundamental approach would be to implement surface treatment techniques that discourage dust adhesion to surfaces in the first place. Acoustic [16], electric [17] and magnetic [18], [19] properties have been associated with the Mars regolith and these properties have motivated investigation of mechanism(s) for lowering dust accumulation. For example, data collected by the Mars rovers has aided in the design of the magnetic arrangements on the Phoenix Lander used to prevent dust from settling on calibration targets [17]. Additionally, thin conductive films have provided relief [3] from excessive amounts of electrostatically driven dust buildup in the past.

Both mechanisms mentioned above have limitations. For example, to create sufficiently strong magnetic fields to be effective as a dust “repellant” technique it would require large and consequently heavy structures due to nature of ferromagnetic materials. This will not be practical for protection of large surface areas. On the other hand, thin film coatings may not be possible for all surfaces particularly if large areas need to be coated.

Surface modification of non-conducting polymers such as silicone rubber with plasma treatment is a commonly practiced and well-studied area [20], [21], [22], [23], [24], [25], [26], [27], [28], [29], [30], [31], [32]. Various gases such as argon [20], [21], [22], nitrogen [20], [21], and oxygen [20], [21] have been used for the modification process and the choice of the gas used to create the plasma depends on the specific application and desired outcomes. Surface plasma treatment of polymers is used mainly to promote surface adhesion to hydrophobic surfaces by rendering the surfaces hydrophilic [33], [34].

The plasma treatment is expected to affect other surface properties such as surface potential of the treated material. For dielectric materials this can be associated with surface charge [35], [36]. However, the effect of such treatments on the surface potential and consequently surface charge have not been studied, in particular, in conjunction with adhesion and attachment of subsequent layers such as dust and regolith. The charging of these polymers could occur through a wide range of interactions, handling and processing that are often inevitable. Therefore, a final “discharging” of the samples is a necessary step that can be performed by means of a non-invasive, inexpensive and simple method using accessible laboratory equipment such as a plasma chamber

In this work, the effect of Ar plasma on surface properties of three popular silicones RTV 655, RTV 615, and Sylgard 184 was investigated. Surfaces were treated with Ar plasma for a variety of time periods under laboratory conditions. The surface potential and static contact angle of each material was compared before and after plasma exposure for each treatment time. Samples were also exposed to Mars dust chamber containing simulant dust JSC-Mars 1 in order to assess the effectiveness of the treatment in lowering the amount of dust captured on each polymer surface. The effect of ultraviolet radiation on the surface properties of RTV 655 was also evaluated and compared with results obtained from exposure to Ar plasma.

## Materials and Methods

### 1. Preparation of RTV 655, RTV 615, and Sylgard 184 samples

A two-component supply of RTV 655 and RTV 615 (Momentive Performance Materials, Columbus, OH) and Sylgard 184 (Dow Corning, Midland, MI) containing an elastomeric pre-polymer (A) and cross-linker (B) was purchased and mixed at a recommended ratio of 10∶1 (pre-polymer to cross linker). The components were mixed thoroughly for 3 min and outgassed in a Precision Scientific Model 19 vacuum oven at room temperature. After complete outgassing they were poured into 4 cm×4 cm aluminum molds, outgassed one more time, and finally cured at 90°C for 1 hr as recommended by the manufacturers. The samples were then removed from their molds, cut into 1 cm×1 cm squares, weighed on an Ohaus Pioneer analytical balance and stored in petri dishes until use. From each polymer type control pieces were kept separately. All samples were made to the same size and thickness 3 mm. The uniformity of the sample thickness was checked before curing for each polymer sample. Multiple batches were made one for the surface potential measurements, one for the contact angle measurements and one for exposure to the dust chamber. The surface potential, contact angle, and dust adhesion properties of each batch was assessed prior to Ar plasma treatments in order to establish the baseline values for each polymer type.

### 2. Surface modification techniques

**Ar Plasma treatment:** Samples of RTV 655, RTV 615, and Sylgard 184 prepared as described formerly were inserted in a Harrick plasma chamber for the Ar plasma treatment. The RF level was set to five and samples were exposed to the Ar plasma for 30, 45, 60, 90, 105, 120, 135, 300, 400, and 600 sec. The exposure time was counted from the time the plasma had struck. After each Ar plasma treatment period, the samples from each type of polymer were transferred to the next necessary experimental setup cued within minutes. These stages included 1) surface potential measurement by means of a Kelvin probe, 2) contact angle measurement, and 3) exposure to a custom built Mars simulant dust chamber. Samples were investigated by means of optical techniques after each exposure time and compared with the control samples.

**UV treatment of samples:** Samples of RTV655 were exposed to a 350 watt Hg lamp with an Oriel light source. The samples were irradiated under atmospheric conditions and at room temperature, all set at a distance of 5 cm away from the lamp housing. Samples were UV irradiated for 1, 5, 12, and 24 consecutive hours. The contact angle and surface potential values of all samples were measured for each exposure time and will be described in the coming sections.

### 3. Surface characterization techniques

**Contact angle measurements:** Static contact angles were measured by means of a VCA Optima XE contact angle measurement unit. A 2 µl drop of deionised (DI) water was deposited on the sample surfaces by a calibrated micro syringe. The receding contact angle was measured for each sample using the VCA Optima software before and after each type of treatment and surface treatment period. For each exposure method and for each exposure time interval at least one polymer sample was kept for contact angle measurements. Samples that were used for contact angle measurement were not used for surface potential measurements, or, exposure to dust chamber since the surface has been modified as a result of exposure to liquid. Measurements were taken at room temperature and atmospheric pressure at all times. Contact angle measurements were taken on at least three different points from each polymer to keep check on local surface variations if any.

**Surface potential measurements:** The surface potential of all samples was measured by means of a Dek Tak 325 electrostatic voltmeter with a 9 mm×9 mm vibrating probe head utilizing the field nullifying technique [39]. Samples were placed on a grounded conducting plate on top of a manual micro-stage with x-y-z motion control. The separation between the probe and the stage was set such that with the samples added, the separation was 0.3 mm, as recommended by the manufacturer in order to minimize the fringing field. The surface potential readings were independent of the height of the probe as determined experimentally and were confirmed theoretically. The probe, sample, and stage were housed inside a custom built Faraday cage. Surface potential readings of each sample were taken by raster scanning the surface in increments of 1 mm taking a reading after the meter reached a stable reading. Care was taken to ensure that the probe area was always directly above a polymer section and away from other material surfaces. Measurements were taken a) before and after Ar plasma exposure for each exposure time interval, and b) before and after vacuum storage.

### 4. Exposure to Mars simulant dust

Samples were exposed to mars simulant dust JSC-1 following the method described previously [3]. All samples were weighed before and after exposure to dust chamber on a Pioneer Ohaus microbalance.

### 5. Recovery of surface properties

In order to assess the recovery rate of the surface potential and surface wettability samples of RTV 655, RTV 615, and Sylgard 184 were exposed to 30 sec of Ar plasma treatment after which surfaces were completely wetting and had surface potential values at or very close to zero. Half of the samples were under ambient conditions in a covered petri dish, while the other half was placed in a Precision Scientific Model 19 vacuum oven and kept at room temperature, but under vacuum and the effect of vacuum storage was studied. The surface potential and contact angle of all sets of samples was measured 12, 24, 36, and 48 hrs post plasma treatment respectively as described in earlier sections.

## Results and Discussion

### 1. Effect of Ar plasma treatment on surface wettability of RTV 655, RTV 615, and Sylgard 184

**Figure 1:**
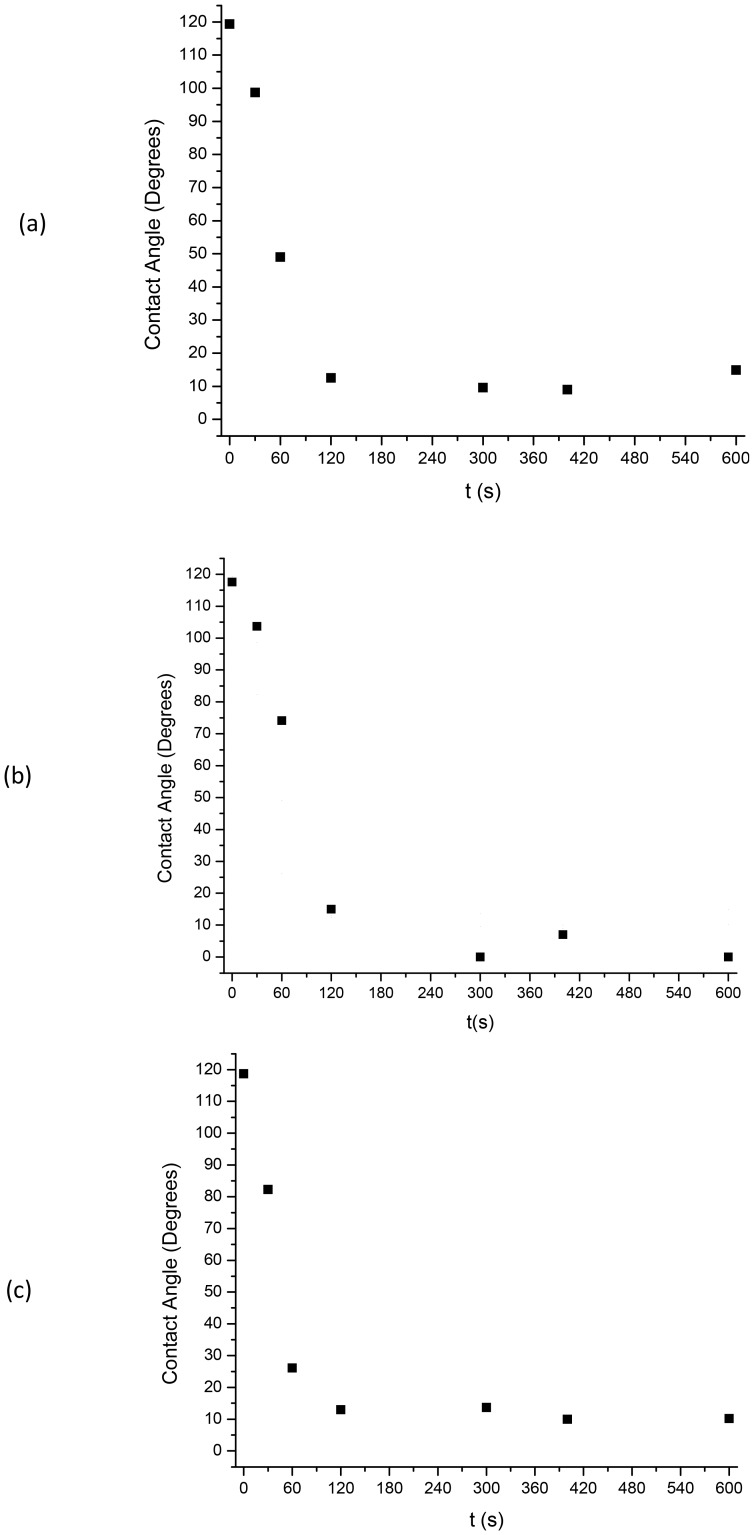
Contact angle versus Ar plasma exposure time. Effect of Ar plasma treatment time on wettability of a) RTV 615, b) RTV 655, and c) Sylgard 184 polymer samples. Prior to any plasma exposure (t = 0 sec) all three polymers show strong hydrophobicity. After 60 sec of Ar plasma treatment contact angles have dropped by at least 50%. All three polymer types are completely wetting at t = 120 sec and beyond.

Samples from each polymer type were exposed to the Ar plasma chamber that is routinely used for cleaning of surfaces. Contact angle measurements performed prior to plasma treatment demonstrated a hydrophobic nature for all three polymer types, as expected. After exposure time of ∼90 sec the surfaces of all three polymers were rendered hydrophilic as shown in Figure 1. As the plasma exposure time was increased the surface contact angle was reduced for RTV 615 (Figure 1a), RTV 655 (Figure 1b), and Sylgard 184 (Figure 1c), until a completely wetting surface was created. Changing of polymer surfaces from hydrophobic to hydrophilic can be related to the flexibility of the siloxane chains which over time re-orient themselves away from the surface of the material and the low molecular weight polydimethylsiloxane from within the bulk of the material will diffuse to the surface [37], [38].

### 2. Effect of Ar plasma treatment on surface potential of RTV 655, RTV 615, and Sylgard 184

**Figure 2:**
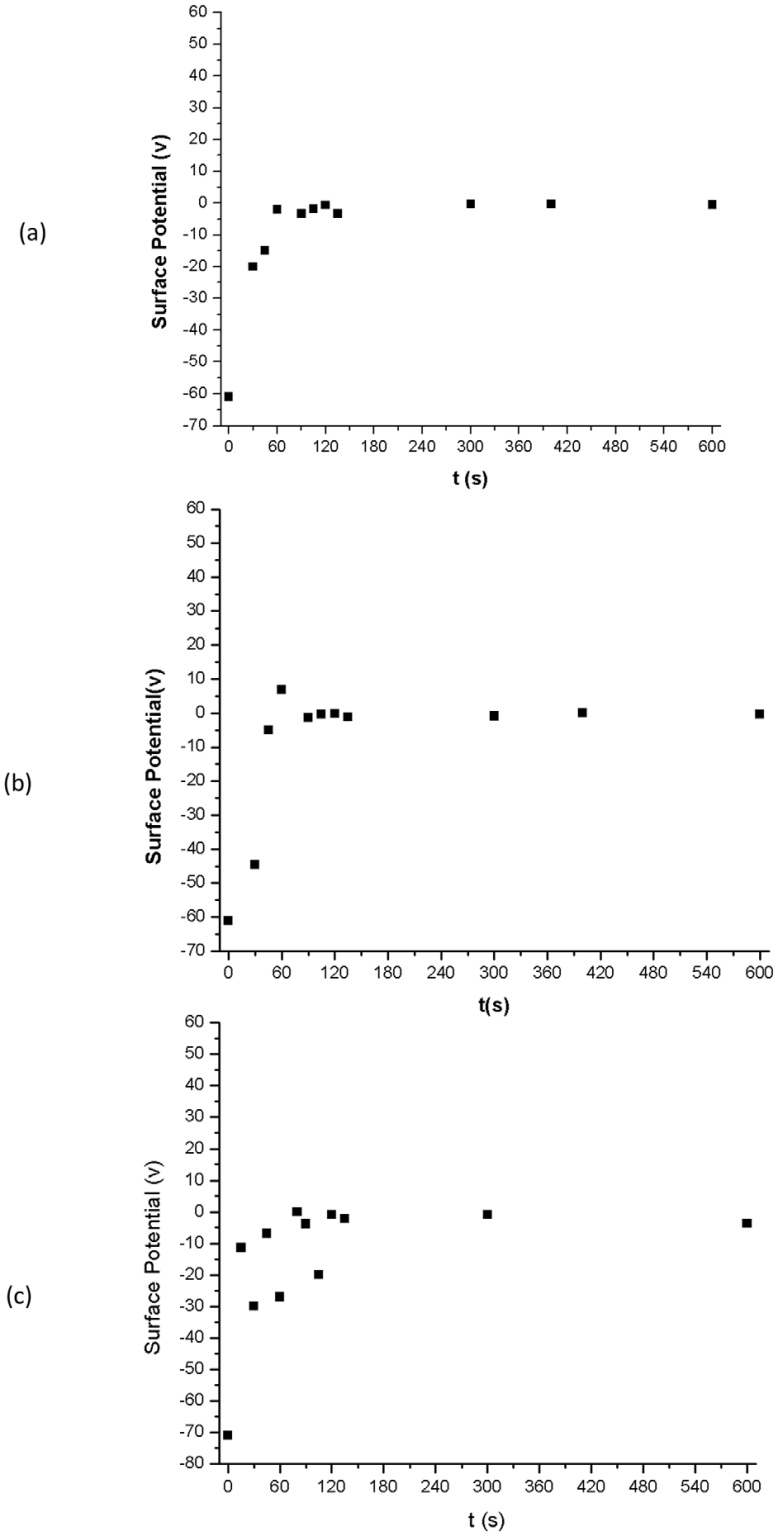
Surface potential versus Ar plasma exposure time. Effect of Ar plasma exposure time on surface potential of a) RTV 655, b) RTV 615, and c) Sylgard 184 measured at room temperature. All three polymers display large negative potentials at t = 0 sec. As the Ar plasma exposure time is increased the surface potential readings decrease and after only 90 sec of exposure time the surface potential values for all three polymers approaches zero.

The effect of the Ar plasma treatment time on surface potential values of the three chosen polymers is shown in Figure 2 for RTV 655, RTV 615, and Sylgard 184. Prior to any plasma treatment the polymer surfaces demonstrated large negative values which are likely to be due to the handling and processing steps that the samples must go through. After exposure to Ar plasma however, the values measured decreased significantly as the exposure time was increased until the surface potential values approached a zero reading as shown in Figures 2a, 2b, and 2c. This trend appeared to be independent of the polymer type and the initial value of surface potential prior to any plasma treatment.

### 3. Effect of Ar plasma treatment on Surface charge density

**Figure 3:**
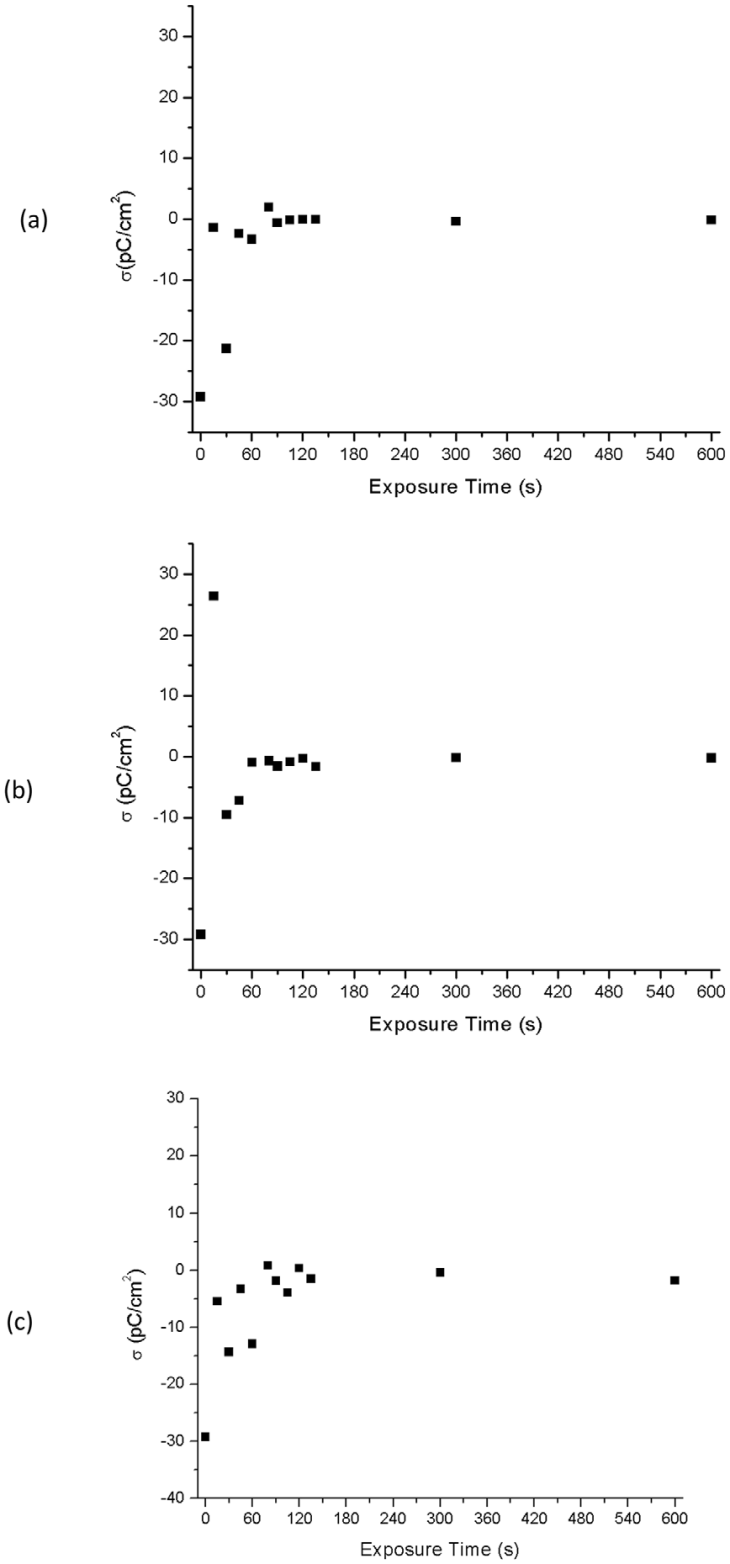
Effect of Ar plasma on surface charge density. Plot of surface charge density (calculated from surface potential values) as a function of Ar plasma exposure time for a) RTV 655, b) RTV 615, and c) Sylgard 184. As the Ar plasma treatment time increases the surface charge values approach zero.

The surface charge density for each polymer type was calculated from the values presented in Figures 2a, 2b, and 2c for each plasma exposure time using equation (1) [39]. The probe-surface system is modeled as a parallel plate capacitor
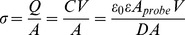
(1)where C is the capacitance of the sample, A is the area of the sample under the probe, Q is the surface charge, V is the measured potential at the surface of the sample, ε is the relative permittivity of the sample (assumed to be 2.7 for RTV 655, RTV 615, Sylgard 184) [40], ε_0_ is the permittivity of free space (8.85×10^−12^ F/m), A_probe_ is the surface area of the probe head (A≈A_probe_), and D is the thickness of the sample under test. Using this simple electrostatic model the surface charge density for each treatment time was calculated and is presented in Figure 3. Figure 3a shows the dependency of surface charge density of RTV 655 as a function of Ar plasma treatment time. The calculated surface charge is significantly high prior to any plasma treatments and drops off rapidly to values very close to zero after a short period of treatment of approximately 50 sec. A similar trend is observed for RTV 615 as seen in Figure 3b where within the first 60 sec the surface charge density decreases from a large negative value of −30 pC/cm^2^ to zero. The behavior of surface charge density for Sylgard 184 is shown in Figure 3c and shows the same trend as was observed for RTV 655, and RTV 615. In all three polymers, the surface charge is essentially nullified as a result of the Ar plasma treatment.

### 4. Effect of Ar plasma treatment on dust adhesion to RTV 655, RTV 615, and Sylgard 184

**Figure 4:**
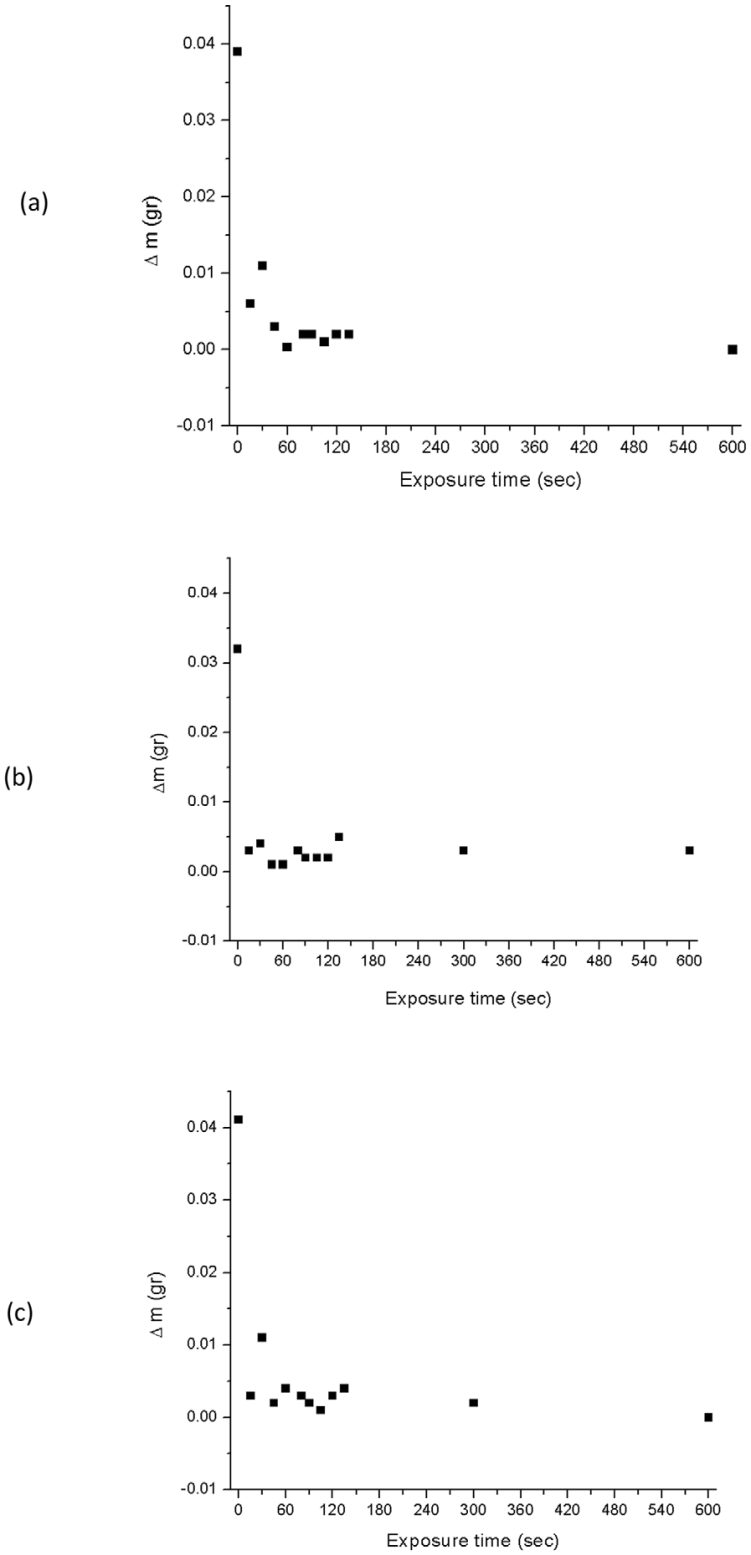
Dust capture versus Ar plasma treatment time. Gravimetric analysis of the effect of Ar plasma treatment time on dust capture for a) RTV 655, b) RTV 615, and c) Sylgard 184. In all three cases a reduction in surface charge by means of Ar plasma has a major impact on dust accumulation and adhesion behavior of PDMS-based polymers.

**Figure 5:**
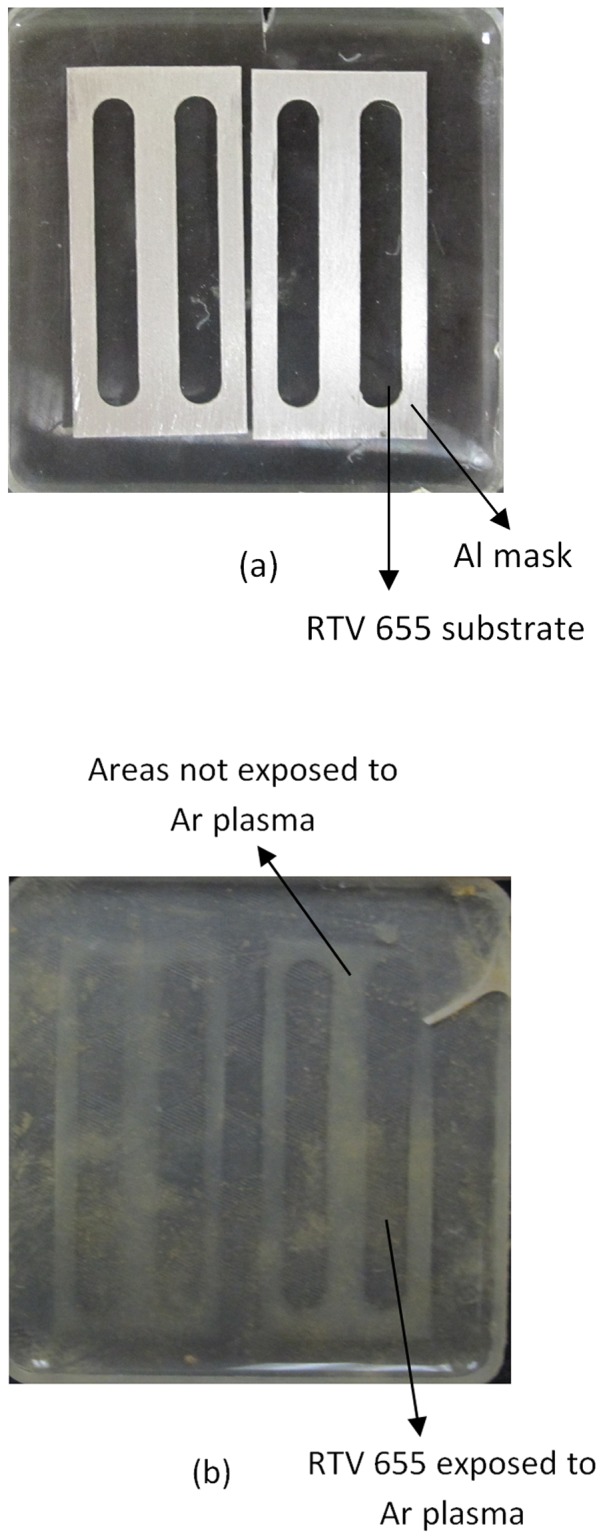
Ar plasma exposure of RTV 655 with mask. Argon plasma exposure of RTV 655 sample with a patterned mask (a) followed by the dust chamber exposure step. The mask pattern can be seen clearly (b) highlighting the impact of the Ar plasma treatment step on the dust adhesion behavior of the polymer.

The effect of Ar plasma treatment time was also assessed on the dust adhesion properties for each polymer type. The amount of dust adhered to each polymer surface after each exposure time was compared with the dust adhesion amount before exposure and represented by Δm (m_before_-m_after_). The results are presented in Figures 4a, 4b, and 4c for RTV 655, RTV 615, and Sylgard 184 respectively where the net amount of dust accumulated (Δm) is measured as a function of plasma treatment time. For all three polymers virtually no detectable amounts of dust is accumulated on the samples surfaces for exposure times greater than 60 sec. This result confirms that the primary cause of dust accumulation on these surfaces is through electrostatic and coulombic interaction and if non-invasive surface treatments are implemented the dust adhesion can be virtually eliminated. The effect of the Ar plasma treatment on dust adhesion is particularly clear in Figure 5 where the surface of RTV 655 has been covered with an Al mask during the plasma treatment and subsequent exposure to Mars dust chamber shows the pattern of the mask outlined as can be seen in Figure 5b.

All polymer surfaces were investigated by means of a Nikon Optiphot upright microscope as well as a Veeco Metrology Dimension 3100 AFM after plasma treatments and no damage to the polymer surfaces was detected after 600 sec of plasma exposure.

### 5. Recovery of surface properties

**Figure 6:**
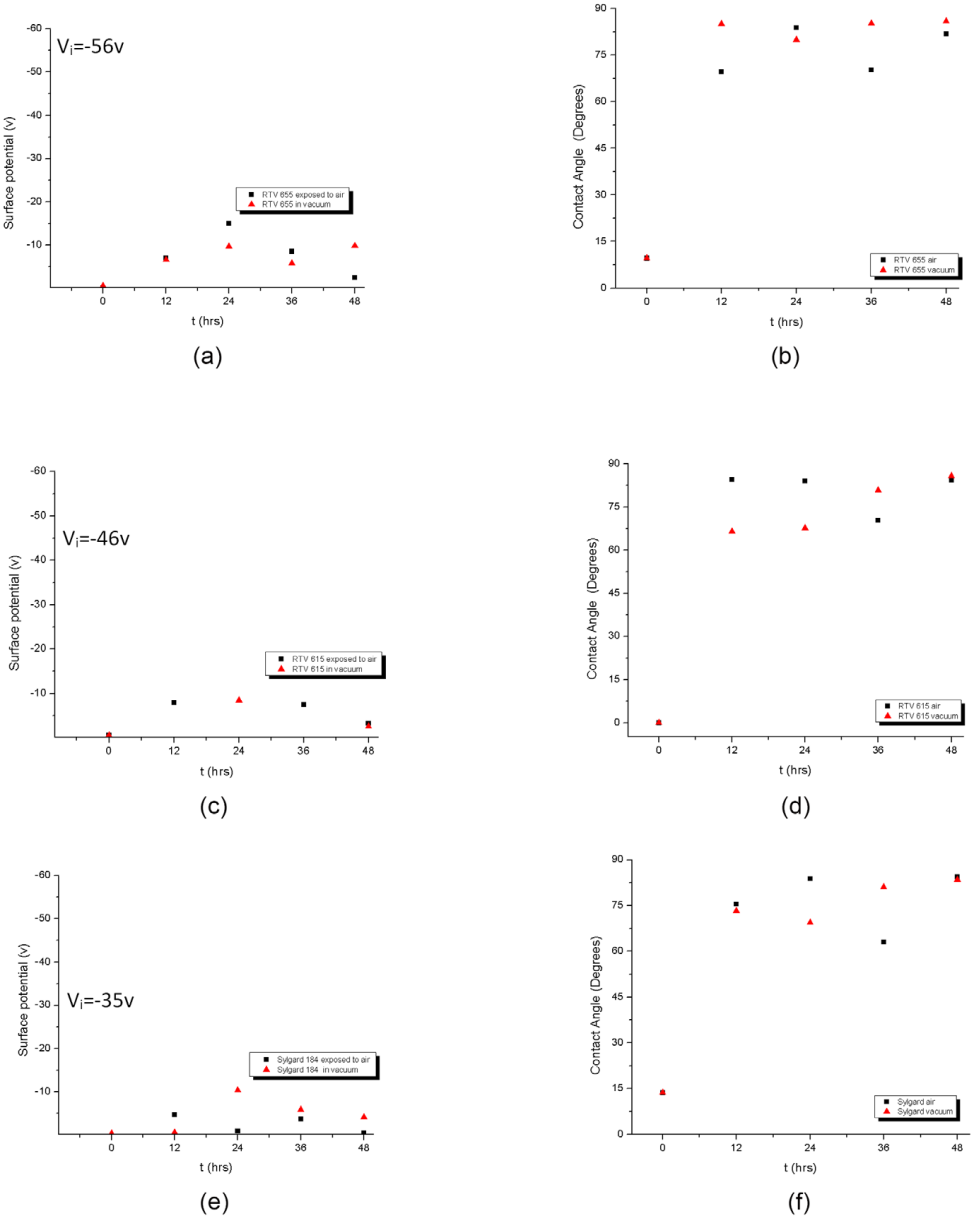
Post Ar plasma treatment recovery. Monitoring recovery of surface potential (a, c, e) and contact angle (b, d, f) after 60 sec Ar plasma treatment for RTV 655, RTV 615, and Sylgard 184. Recovery conditions under vacuum and at atmospheric pressure were also compared. The surface hydrophobicity of all three polymers recovered within the first twelve hours while the surface potential remained at values close to zero even 48 hrs after treatment. Recovery rate appears to be independent of the storage conditions of vacuum versus ambient.

An important part of this investigation was monitoring the recovery of the effected surface properties as a function of time and storage conditions. Samples of RTV 655, RTV 615, and Sylgard 184 post plasma treatment were stored under atmospheric and vacuum conditions and the surface potential and surface contact angle was measured every twelve hours for forty eight hours post plasma treatment (see Figure 6) and then measured three months after treatment time (data not shown). Over time, the surface wettability reduced and all three polymers reverted to their original hydrophobic nature (Figure 6b, 6d, 6e) regardless of the storage conditions. The surface potential however remained at very low values compared to their initial values prior to any plasma treatment as listed for each polymer in Figures 6a, 6c, and 6d on each graph.

### 6. Effect of UV radiation on polymers

**Figure 7:**
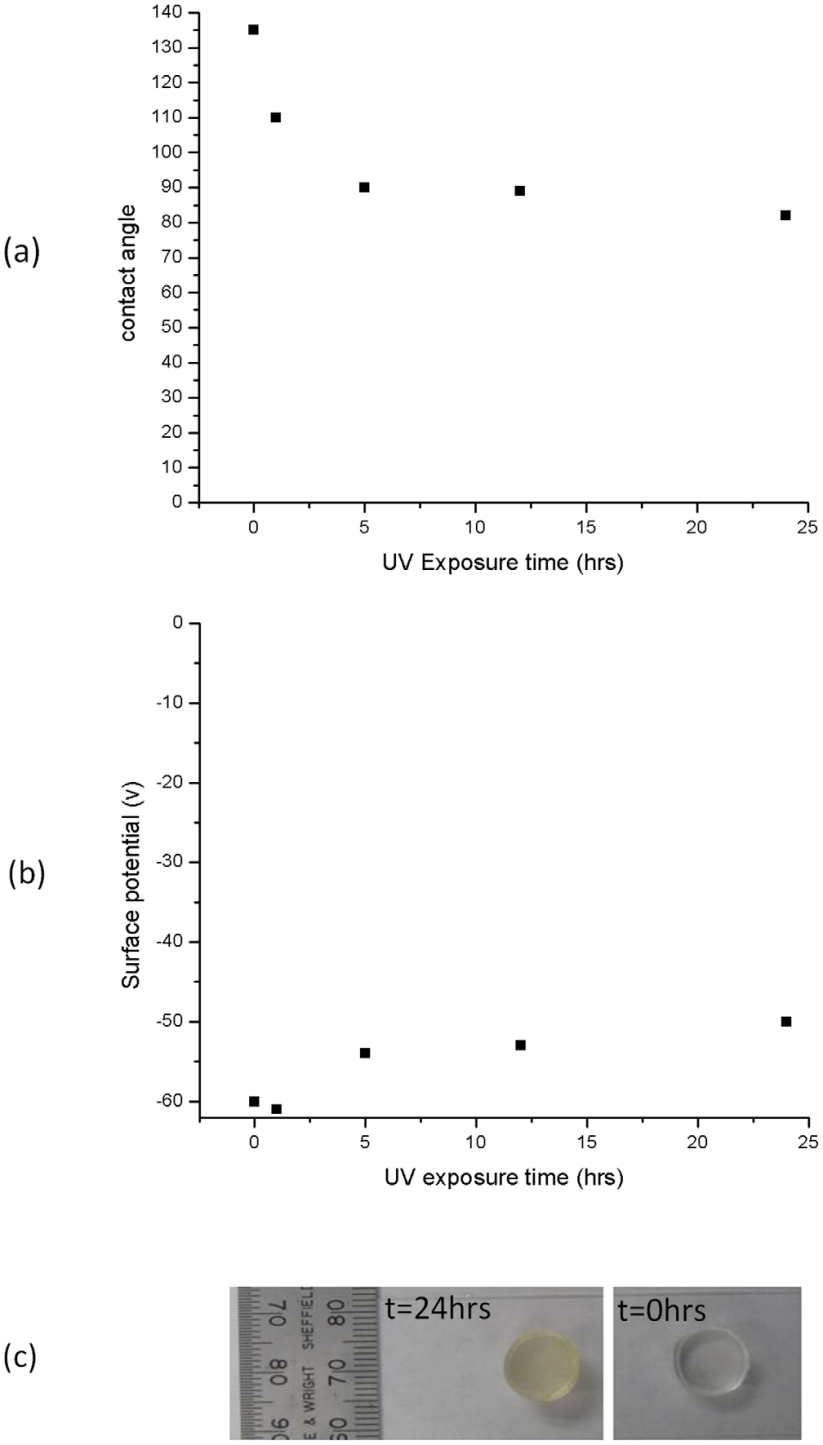
Effect of UV on RTV 655. Effect of UV radiation on a) contact angle and b) surface potential of RTV 655. Exposure to UV radiation has little impact on the surface wettability and on the surface potential of RTV 655 after 24 hrs irradiation. Dust adhesion to the UV-treated surfaces drops significantly after a long exposure time. Figure (c) shows an image of the damage caused to the polymer sample after 24 hrs of UV irradiation.

Next the effect of ultraviolet (UV) radiation on the surface potential and the wettability of RTV 655 was investigated and compared to the effect of Ar plasma treatment. In Figure 7a the effect of UV radiation on the surface wettability of RTV 655 is shown and it is clear that UV exposure has a less significant effect on surface wettability than Ar plasma exposure, despite the prolonged exposure time. The surface potential of RTV 655 remains close to its original values despite 24 hrs of continuous radiation exposure as shown in Figure 7b. Ultraviolet radiation does however have a degrading effect on the exposed polymer as can be seen in Figure 7c where a RTV 655 samples is visually compared before and after 24 hrs of UV irradiation under atmospheric conditions. Note the visible change in the surface of the material the UV radiation has caused the sample.

## Summary

Ar plasma surface treatment was used to modify the surface properties of three commonly-used RTV-based polymers RTV 655, RTV 615, and Sylgard 184. Initially all surfaces were super hydrophobic and demonstrated large values of surface potential, in excess of −58 V. After brief plasma exposure the surfaces were rendered hydrophilic and the surface potentials dropped to zero volts or values very close to zero, regardless of the initial surface potential values prior to any plasma treatment. Argon plasma exposure of the polymers resulted in a charge-free surface to which no simulant dust adhered. Therefore, Ar plasma treatment effectively removed the surface charge preexistent on the polymer surfaces and consequently eliminated dust adhesion and attachment. The effect of UV irradiation was minimal on the surface charge, it did however, cause severe surface discoloration and is clearly having an adverse effect of the polymeric material. Additionally, monitoring the recovery rate of polymer properties demonstrated that if polymer surfaces are kept isolated surface charge will remain zero even though surface hydrophobicity will return.
