# Correction: Effect of Surface Plasma Treatments on the Adhesion of Mars JSC 1 Simulant Dust to RTV 655, RTV 615, and Sylgard 184

**DOI:** 10.1371/annotation/4e090015-ac60-4afc-b4a3-e2008063bdec

**Published:** 2012-11-26

**Authors:** Firouzeh Sabri, Jeffrey G. Marchetta, M. Sinden-Redding, James J. Habenicht, Thien Phung Chung, Charles N. Melton, Chris J. Hatch, Robert L. Lirette

The fifth author's name was spelled incorrectly. The correct name is: Thien-Chuong N Phung.

The correct citation is: Sabri F, Marchetta JG, Sinden-Redding M, Habenicht JJ, Phung T-CN, et al. (2012) Effect of Surface Plasma Treatments on the Adhesion of Mars JSC 1 Simulant Dust to RTV 655, RTV 615, and Sylgard 184. PLoS ONE 7(10): e45719. doi:10.1371/journal.pone.0045719

